# Identification of lncRNA NR_028138.1 as a biomarker and construction of a ceRNA network for bipolar disorder

**DOI:** 10.1038/s41598-021-94122-7

**Published:** 2021-08-02

**Authors:** Ling He, Pengtao Zou, Wanlei Sun, Yonghui Fu, Wenfeng He, Juxiang Li

**Affiliations:** 1 Department of Cardiovascular Medicine, Second Affiliated Hospital of Nanchang University, Minde Road No. 1, Nanchang, 330006 Jiangxi China; 2 Department of Psychiatry, Jiangxi Mental Hospital, Nanchang, 330029 Jiangxi China; 3 Department of Clinical Laboratory, Second Affiliated Hospital of Nanchang University, Nanchang, 330006 China; 4 Jiangxi Key Laboratory of Molecular Medicine, Second Affiliated Hospital of Nanchang University, Minde Road No. 1, Nanchang, 330006 Jiangxi China

**Keywords:** Computational biology and bioinformatics, Neuroscience

## Abstract

The pathogenesis of bipolar disorder (BD), a chronic mood disorder, is largely unknown. Noncoding RNAs play important roles in the pathogenesis of BD. However, little is known about the correlations of long noncoding RNAs (lncRNAs) with BD. Illumina high-throughput sequencing in BD patients and normal controls was used to identify differentially expressed (DE) genes. Two-step real-time quantitative reverse transcription polymerase chain reaction (qRT-PCR) was used to validate DE-RNAs in the first cohort (50 BD and 50 control subjects). Gene Ontology (GO) and Kyoto Encyclopedia of Genes and Genomes (KEGG) pathways and lncRNA-mRNA coexpression and lncRNA-microRNA (miRNA)-messenger RNA (mRNA) competing endogenous RNA (ceRNA) network analyses were used to predict the functions of DE-RNAs. Receiver operating characteristic (ROC) curve analysis and logistic regression were applied to evaluate diagnostic performance in an additional testing group (80 BD and 66 control subjects). A total of 576 significantly DE-lncRNAs and 262 DE-mRNAs were identified in BD patients, and 95 lncRNA-miRNA-mRNA interactions were used to construct a ceRNA regulatory network. Analysis of the first cohort showed that six RNAs (NR_028138.1, TCONS_00018621, TCONS_00002186, TNF, PID1, and SDK1) were differentially expressed in the BD group (*P* < 0.01). NR_028138.1 was used to establish a BD diagnostic model (area under the ROC curve 0.923, *P* < 0.004, 95% CI: 0.830–0.999). Verification in the second cohort revealed uniformly significant differences in NR_028138.1 (*P* < 0.0001). This study constructed a ceRNA regulatory network and provided a hypothesis for the pathogenesis of BD. NR_028138.1 was identified as a central element involved in the transcriptional regulation in BD and a potential biomarker.

## Introduction

Bipolar disorder (BD) is a mood disorder caused by genetic and environmental factors^1^. The incidence rate of BD in China has increased up to 2%, and the number of people with BD has reached more than 8 million. BD is characterized by complex clinical manifestations, difficult treatment, poor prognosis, and high risk of suicide^2^. Therefore, BD has become a critical social public health issue that needs to be addressed. It is urgent to explore the biomarkers that are able to predict the key factors important for BD occurrence and therapy.

Accumulating evidence indicates that long noncoding RNAs (lncRNAs) participate in many central nervous system diseases (CNDs), acting as pathogenic factors, potential therapeutic targets, prognostic indicators, or biomarkers^3–6^. Polesskaya et al. demonstrated in 2003 that lncRNA psza11q14 is associated with the pathogenesis of schizophrenia detected by autopsy of the brain tissue. The expression of psza11q14 in patients with schizophrenia is significantly decreased compared with that in the control subjects. Subsequently, the authors confirmed that psza11q14 expression is decreased in Brodmann areas 21, 22, and 9 and in the hippocampus in 36 schizophrenic patients compared with that in 35 normal control subjects. Functional experiments showed that psza11q14 is located on 11q14 within the first intron region of the dlg-2 gene and is transcribed in the opposite direction to dlg-2; moreover, dlg-2 plays an important role in the regulation of the N-methyl-D-aspartate (NMDA) receptor^7^. Issler et al. demonstrated that LINC00473 is a potential risk factor for enhanced susceptibility to depression in females and that LINC00473 expression is downregulated in the prefrontal cortex (PFC) in females but not in males^8^. LINC00473 is related to 35 protein-coding genes, most of which are associated with depression. However, the expression patterns and functions of lncRNAs in the context of BD remain largely unknown.

Several recent studies demonstrated that lncRNAs competitively bind to microRNAs (miRNAs) to form a competing endogenous RNA (ceRNA) network^9–12^, and a novel regulatory mode of the network is composed of ceRNA-miRNA-mRNA interactions. The regulatory mechanisms of ceRNAs play an important role in the pathogenesis of CNDs^13,14^. Wang et al. reported that lncRNA WT1-AS suppresses oxidative stress injury and apoptosis in Alzheimer's disease via inhibition of the miR-375/SIX4 axis^15^. However, only a few studies investigated ceRNA networks in BD.

Therefore, this study aimed to investigate differentially expressed lncRNAs (DE-lncRNAs) in BD patients versus control subjects and comprehensively analyze a lncRNA-miRNA-mRNA ceRNA network involved in BD. We also evaluated whether identified lncRNAs are promising peripheral biomarkers. These findings may provide potential targets for the development of novel diagnostic and therapeutic strategies against BD.

## Materials and methods

### Collection of patient samples

This study was approved by the Ethics Committee of the Second Affiliated Hospital of Nanchang University, China. The recruitment procedure followed relevant guidelines. All donors and their family members gave informed consent for the collection and use of the samples in this study. The subjects were divided into two cohorts. The first cohort was used for RNA sequencing. Four outpatients and inpatients who met the diagnostic criteria of BD were recruited at Jiangxi Psychiatric Hospital (January to December 2017), and four healthy volunteers from the surrounding communities and schools were included as the control subjects. Healthy control subjects did not have past history of psychiatric or neurodegenerative disorders, intellectual disability, or cancer. The second cohort was used for clinical validation. The subjects were recruited similar to the first cohort (January 2017 to December 2020). A total of 130 BD patients and 116 normal control subjects were recruited. The inclusion criteria were as follows. (i) The patients were diagnosed by two psychiatrists based on the Structured Clinical Interview for DSM-IV. (ii) The age of the patients was between 16 and 45 years. (iii) The patients did not receive any psychotropic medications (including antipsychotics, antidepressants, mood stabilizers, and benzodiazepines) for at least four weeks. (iv) Healthy controls did not have any underlying diseases (past history of psychiatric or neurodegenerative disorders, intellectual disability, cancer, or infection).

### RNA isolation and library construction

Total RNA was extracted from peripheral blood using a PAXgene blood RNA kit (Thermo Scientific, USA) according to the manufacturer’s instructions. RNA was quantified using a NanoDrop 2000 spectrophotometer (Thermo Scientific, USA), and the RNA integrity number (RIN) was estimated using an Agilent 2100 bioanalyzer (Agilent Technologies, USA). Samples with RIN ≥ 7 were considered adequate for subsequent analysis. Two micrograms of RNA per sample was used for lncRNA library construction by a TruSeq Stranded Total RNA kit with Ribo-Zero Gold (cat. no. 15021048, Illumina, USA). Briefly, ribosomal RNA was removed using a Ribo-Zero Gold rRNA removal kit (Illumina), and rRNA-free RNA was reverse transcribed into complementary DNA (cDNA). After ligation of the terminal adapters, PCR amplification, and purification, the quality of the library was assessed using an Agilent bioanalyzer 2100 system. Then, the libraries were sequenced on the Illumina sequencing platform (HiSeq X Ten) with PE150 according to the manufacturer’s protocol. All data analysis was performed by OE Biotech Co. Ltd. (Shanghai, China).

### DE-RNA analysis

DE-mRNAs and DE-lncRNAs in the BD group versus the control group were analyzed using DEGseq 2 package^16^. Genes with q-values (false discovery rate (FDR)) lower than 0.05 and fold-changes higher than 2.0 were considered differentially expressed. Volcano plots and heatmaps of DE-RNAs were generated by R software (https://www.r-project.org/).

### Functional annotation and enrichment analysis

Functional annotation of the genes targeted by DE-lncRNAs was performed by Gene Ontology (GO; http://www.geneontology.org) term analysis and Kyoto Encyclopedia of Genes and Genomes (KEGG; www.kegg.jp/kegg/kegg1.html) pathway analysis. Corrected *P*-values < 0.05 indicated significant enrichment by the genes targeted by DE-lncRNA.

### Association analysis of a lncRNA and mRNA coexpression network

DE-lncRNAs and DE-mRNAs were identified based on fold change filtering. The coexpression relationships of each DE-lncRNA and DE-mRNA were calculated using Pearson’s correlation coefficient (PCC). PCC > 0.95 or < -0.95 and *P* value < 0.05 were used to define coexpressed DE-lncRNA—DE-mRNA pairs.

### Construction of a lncRNA-miRNA-mRNA ceRNA network

RNAs in the DE-lncRNA—DE-mRNA coexpression network were used to construct a ceRNA network. A lncRNA-miRNA-mRNA network was generated as follows. First, DE-lncRNAs were input into the miRDB database to predict target miRNAs. Second, target miRNAs were transferred into the miRDB (http://www.mirdb.org/), TarBase V8 (http://microrna.gr/tarbase/), and TargetScan7.1 (http://www.targetscan.org/vert_71/) databases to predict target mRNAs. Third, target mRNAs were intersected with DE-mRNAs in the DE-lncRNA—DE-mRNA coexpression network. Finally, these interactions were combined to construct a lncRNA-miRNA-mRNA ceRNA network by Cytoscape v3.7.1 software (https://cytoscape.org/)^17^.

### Quantitative real-time PCR

Real-time PCR was performed using a PCR thermocycler (ABI 7900TH, USA) and SYBR Green fluorescence dye according to the manufacturers’ protocol. The sequences of lncRNA-specific primer were designed using Primer BLAST (https://www.ncbi.nlm.nih.gov/tools/primer-blast/) and synthesized by TsingKe Biotech. The expression levels of lncRNAs were normalized to the expression levels of the reference β-actin gene and were calculated using the 2^-ΔΔCt^ method^18^.

## Statistical analysis

Statistical calculations were performed using SPSS 23.0 (SPSS, Inc., Chicago, IL, USA), R software, and GraphPad Prism 7(GraphPad Software, inc.), and a value of *P* < 0.05 was considered statistically significant. Demographic and clinical characteristics of the BD and control groups were evaluated by chi-squared test. qPCR data were evaluated by a nonparametric test. Fisher's exact test was used to evaluate the significance of GO terms or enrichment of pathway identifiers in differentially expressed genes. Pearson correlations between lncRNAs and mRNAs were calculated.

Receiver operating characteristic (ROC) curves were used to assess specificity and sensitivity of lncRNAs for differentiation of BD patients from the control subjects based on area under the ROC curve (AUC). The cutoff values for optimal diagnostic points of the ROC curve were calculated at the largest Youden’s index (sensitivity + specificity − 1). Binary logistic regression and ROC curves were used to improve diagnostic efficiency.

## Results

### Analysis of the patient data

The samples of four patients with BD and four control subjects were used for high-throughput sequencing, and 100 (50 BD and 50 control subjects) and 146 (80 BD and 66 control subjects) subjects were used for separate qRT-PCR analyses. Clinical characteristics of all recruited subjects are summarized in Table 1. Age, sex, education, or family history of these two groups were not significantly different (*P* > 0.05).

**Table 1:** Basic demographic and clinical characteristics of BD and healthy controls.

variables		BD(n = 134)	HC (n = 120)	*P*
Age	24.8 ± 6.63		23.5 ± 5.15	0.919
Age range (years)	15–35		18–40	
Gender				0.74
Male	64		63	
Female	70		57	
Education (years)	13.3 ± 4.0		13.87 ± 3.9	0.957
Duration of illness (months)	36.15 ± 42.37		N/A	
Family history (n(%))	39(29.1%)		N/A	
HAMD	32.38 ± 6.04		N/A	
YMRS	24.85 ± 14.11		N/A	

*HC* healthy control, *HAMD* Hamilton depression rating scale, *YMRS* Young manic rating scale, *N/A* not available. *P* < 0.01 was considered statistically significant.

### Identification of differential gene expression

**Figure 1:**
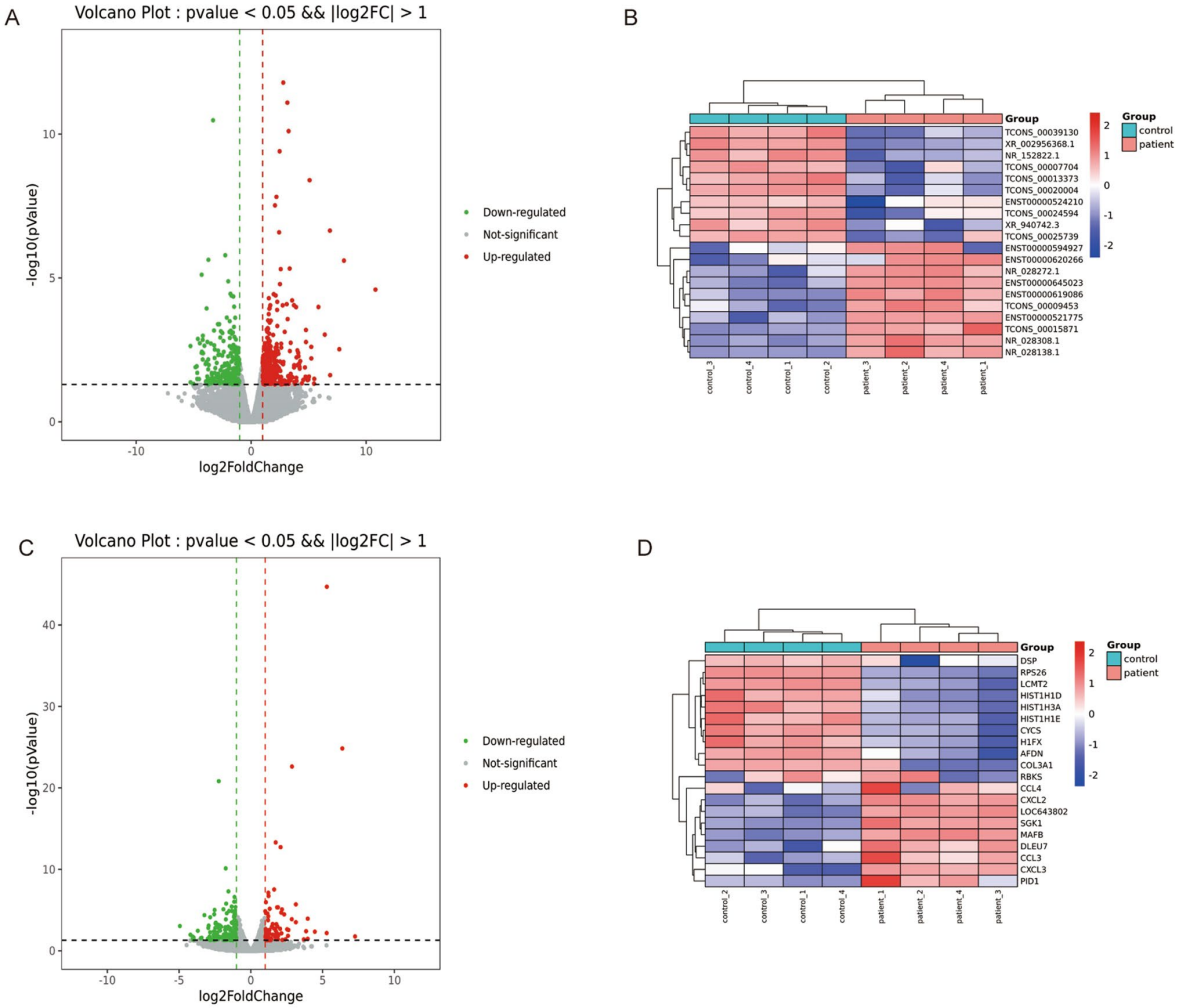
Differentially expressed lncRNAs (DE-lncRNAs) and mRNAs (DE-mRNAs) in the BD versus control groups. (**A**) Volcano plot of DE-lncRNAs in the BD versus control groups. (**B**) Heatmap of DE-lncRNAs in the BD versus control groups. Gray dots represent the genes that are not significantly differentially expressed. Green and red dots indicate significantly upregulated and downregulated genes, respectively. (**C**) Volcano plot of DE-mRNAs in the BD versus control groups. (**D**) Heatmap of DE-mRNAs in the BD versus control groups. Rows and columns represent the genes and samples (|log twofold change|≥ 1.0 and *P* value ≤ 0.05). Volcano plots and heatmaps of DE-RNAs were generated by R software.

We used the DEG-seq R package to determine the differences in the expression levels of lncRNAs and mRNAs between the BD and normal groups. A total of 576 significantly DE-lncRNAs and 262 DE-mRNAs were identified with the threshold of |log_2_FC|≥ 1 and *P*-value ≤ 0.05. A total of 316 DE-lncRNAs and 97 DE-mRNAs were upregulated, and 260 DE-lncRNAs and 165 DE-mRNAs were downregulated. Volcano plot of DE-lncRNAs and DE-mRNAs are shown in Fig. 1A,C. Heatmaps of 20 DE-lncRNAs and DE-mRNAs (BD patient group versus control group) are presented in Fig. 1B,D, including top 10 upregulated and top 10 downregulated lncRNAs.

### Enrichment analysis of DE-lncRNAs

**Figure 2:**
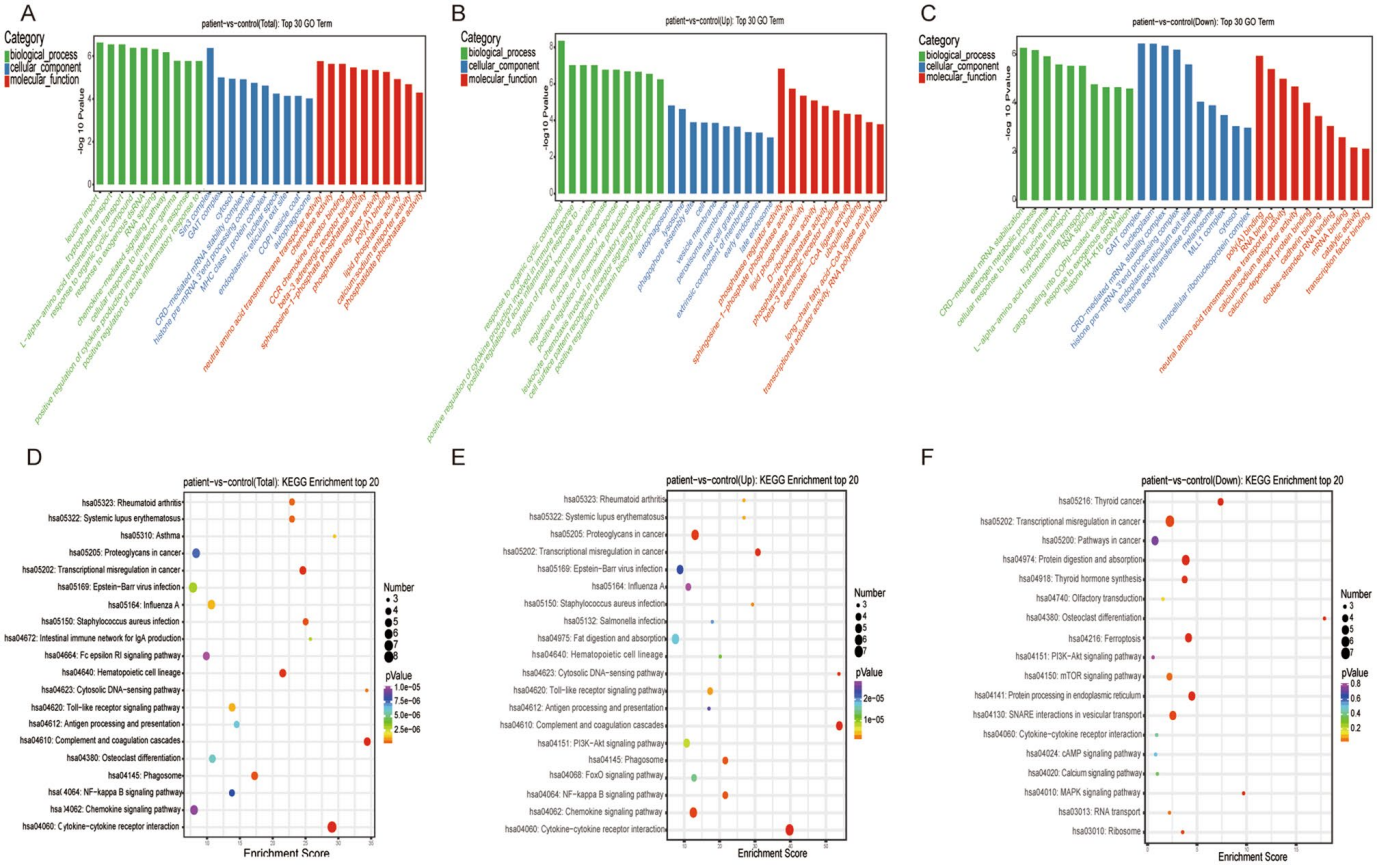
GO term and KEGG pathway enrichment analyses of DE-lncRNAs. (**A**–**C**) Top 30 enriched terms for all, upregulated, and downregulated genes. (**D**–**F**) Top 20 enriched terns for all, upregulated, and downregulated genes. The counts correspond to the numbers of the genes in a GO term or in a KEGG pathway.

GO and KEGG enrichment analyses were used to assess and predict potential functions of identified DE-lncRNAs. Top 30 GO terms are shown in Fig. 2A. Cellular component (CC) analysis indicated that potential target genes were mainly involved in cell junctions, cell parts, and extracellular matrix. Biological process (BP) analysis showed that potential target genes were associated with biological adhesion, biological regulation, cell killing, and cellular component organization or biogenesis. In the molecular function (MF) category, potential target genes were significantly enriched in binding, catalytic activity, and channel regulator activity. Top 30 GO terms enriched in upregulated and downregulated DE-lncRNAs are shown in Fig. 2B,C.

KEGG pathway analysis revealed top 20 pathways related to potential target genes of DE-lncRNAs (Fig. 2D). Upregulated DE-lncRNAs were mainly involved in cytokine-cytokine receptor interactions, proteoglycans in cancer, and chemokine signaling pathway (Fig. 2E). Downregulated DE-lncRNAs were involved in transcriptional dysregulation in cancer, protein digestion and absorption, and ferroptosis (Fig. 2F).

### Construction of a lncRNA-mRNA coexpression network

**Figure 3:**
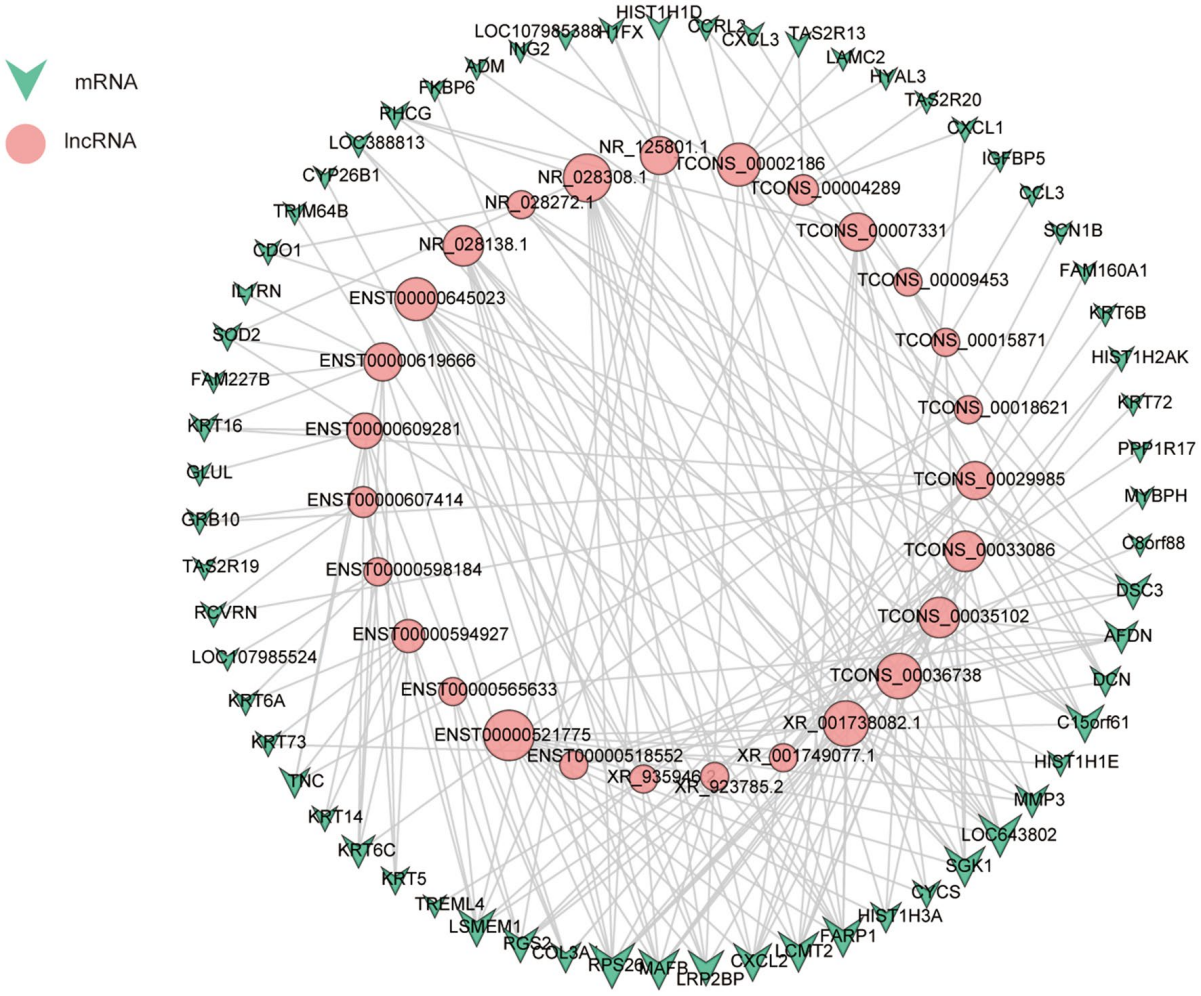
LncRNA-mRNA coexpression network. Pink circular nodes represent lncRNAs, and green triangles represent mRNAs. Gray lines correspond to coexpression relationships between lncRNAs and mRNAs (PCC > 0.95).

A total of 190 coexpression interactions of 27 DE-lncRNAs and 63 DE-mRNAs were included (Fig. 3). All DE-lncRNAs and DE-mRNAs involved in the coexpression network were upregulated in the BD group versus the normal group. ENST00000521775 lncRNA had the highest involvement and participated in 13 interactions of the lncRNA-mRNA pairs. RPS26 mRNA had the highest involvement and was associated with 9 lncRNA-mRNA pairs.

### Construction of a ceRNA network

**Figure 4:**
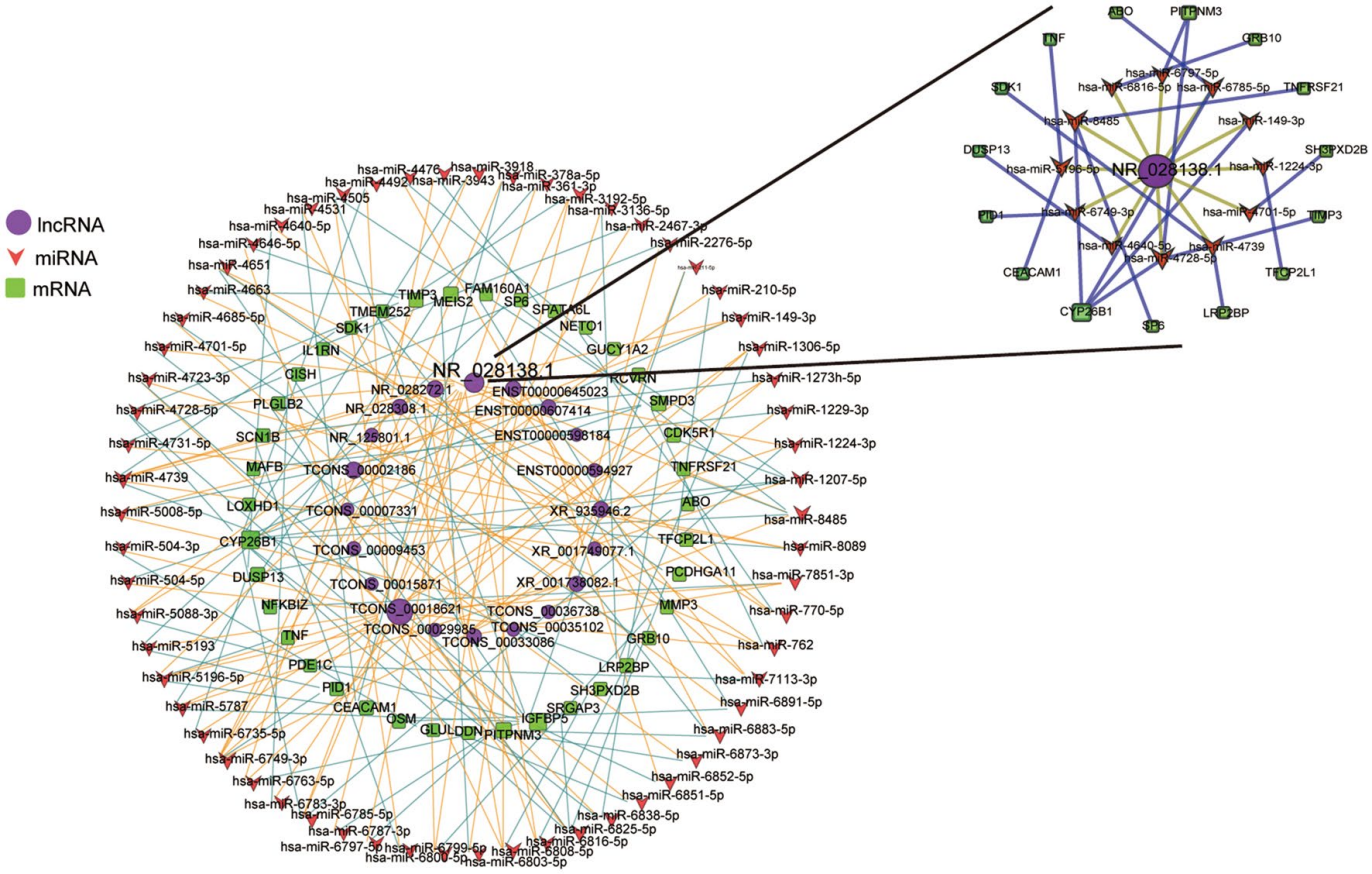
CeRNA network. Red triangles, green squares, and purple circles represent miRNAs, mRNAs, and lncRNAs, respectively. Orange and blue lines represent predicted lncRNA-miRNA and miRNA-mRNA associations, respectively (PCC > 0.95).

Analysis using miRDB identified 95 lncRNA-miRNA interactions (20 lncRNAs and 62 miRNAs), and 62 miRNAs in these pairs were associated with 39 DE-mRNAs in the coexpression network, forming a ceRNA network, which was composed of 39 mRNAs, 20 lncRNAs, and 62 target miRNAs and included 71 lncRNA-miRNA-mRNA ceRNA circular pathways (Fig. 4). TCONS_00018621 lncRNA had the highest involvement in the ceRNA network and participated in 19 ceRNA pathways. CYP26B1 mRNA had the highest involvement and was associated with 12 ceRNA pathways. Furthermore, hsa-miR-4739 miRNA had the highest involvement and was associated with 7 ceRNA pathways.

### Validation of DE-RNAs by qRT-PCR

**Figure 5:**
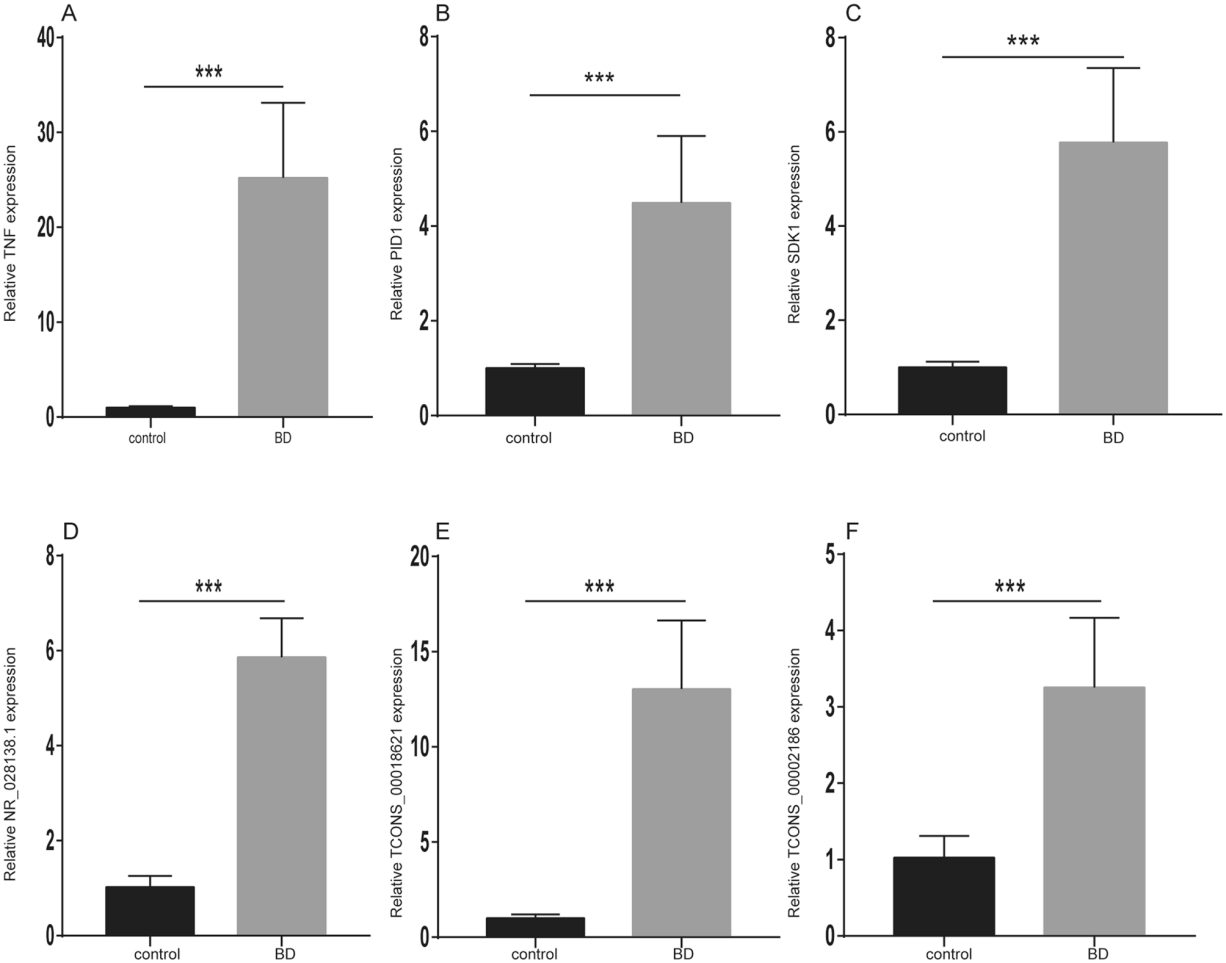
qRT-PCR validation of relative expression levels of six RNAs in BD patients and control subjects. (**A**–**F**) The expression of RNAs (TNF, PID1, SDK1, NR_028138.1, TCONS_00018621, and TCONS_00002186) in the BD and control groups. Black stars represent significant differences.

qRT-PCR analysis of 6 selected DE-RNAs, including 3 lncRNAs with the highest number of interactions in the ceRNA network (NR_028138.1, TCONS_00018621, and TCONS_00002186) and 3 coexpressed mRNAs (TNF, PID1, and SDK1) in the BD and normal groups was used to validate associations between BD and the ceRNA pathways. As shown in Fig. 5, the expression levels of RNAs were upregulated in the BD group compared with those in the normal group, which was consistent with the sequencing results. The differences in the expression of NR_028138.1 (threefold increase versus the normal group) were more pronounced.

### Evaluation of lncRNAs as diagnostic markers for BD

**Figure 6:**
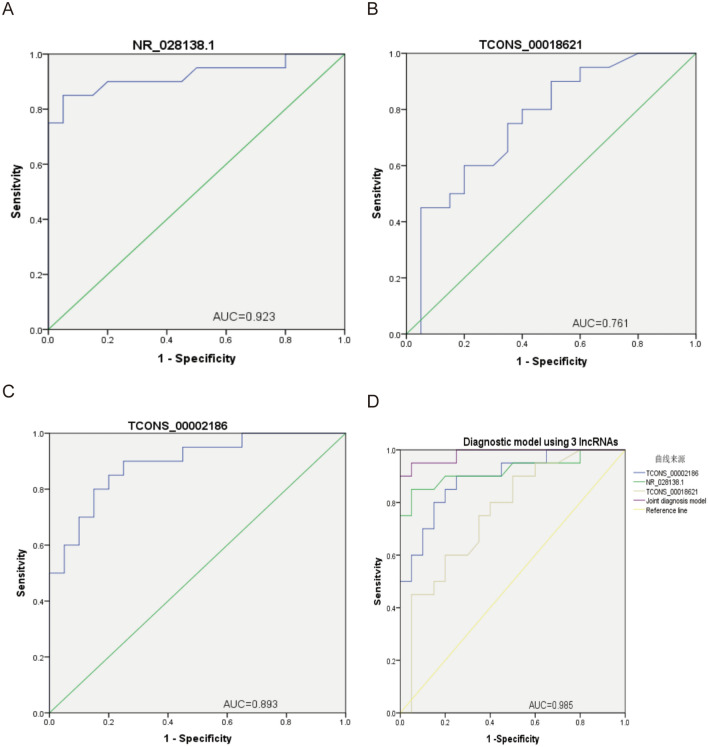
Diagnostic power of the three lncRNA candidates evaluated using receiver operating characteristic (ROC) curves. (**A**) NR_028138.1, (**B**) TCONS_00018621, and (**C**) TCONS_00002186. (**D**) Diagnostic model using 3 lncRNAs. Optimal diagnostic points were considered cutoff values with the largest Youden’s index (sensitivity + specificity – 1).

The prediction probability based on a binary logistic regression model was determined by ROC curve analysis. The diagnostic values for NR_028138.1, TCONS_00018621, TCONS_00002186, and three lncRNA combinations for BD were assessed. As shown in Fig. 6A–C, the AUCs for NR_028138.1, TCONS_00018621, and TCONS_00002186 were 0.923, 0.761, and 0.893, respectively. These 3 lncRNAs were combined to improve the diagnostic power, and a diagnostic model was constructed using binary logistic regression. AUC for the combination of 3 lncRNAs was 0.985 (*P* < 0.0001) (Fig. 6D).

### NR_028138.1 is a potential biomarker for BD

**Figure 7:**
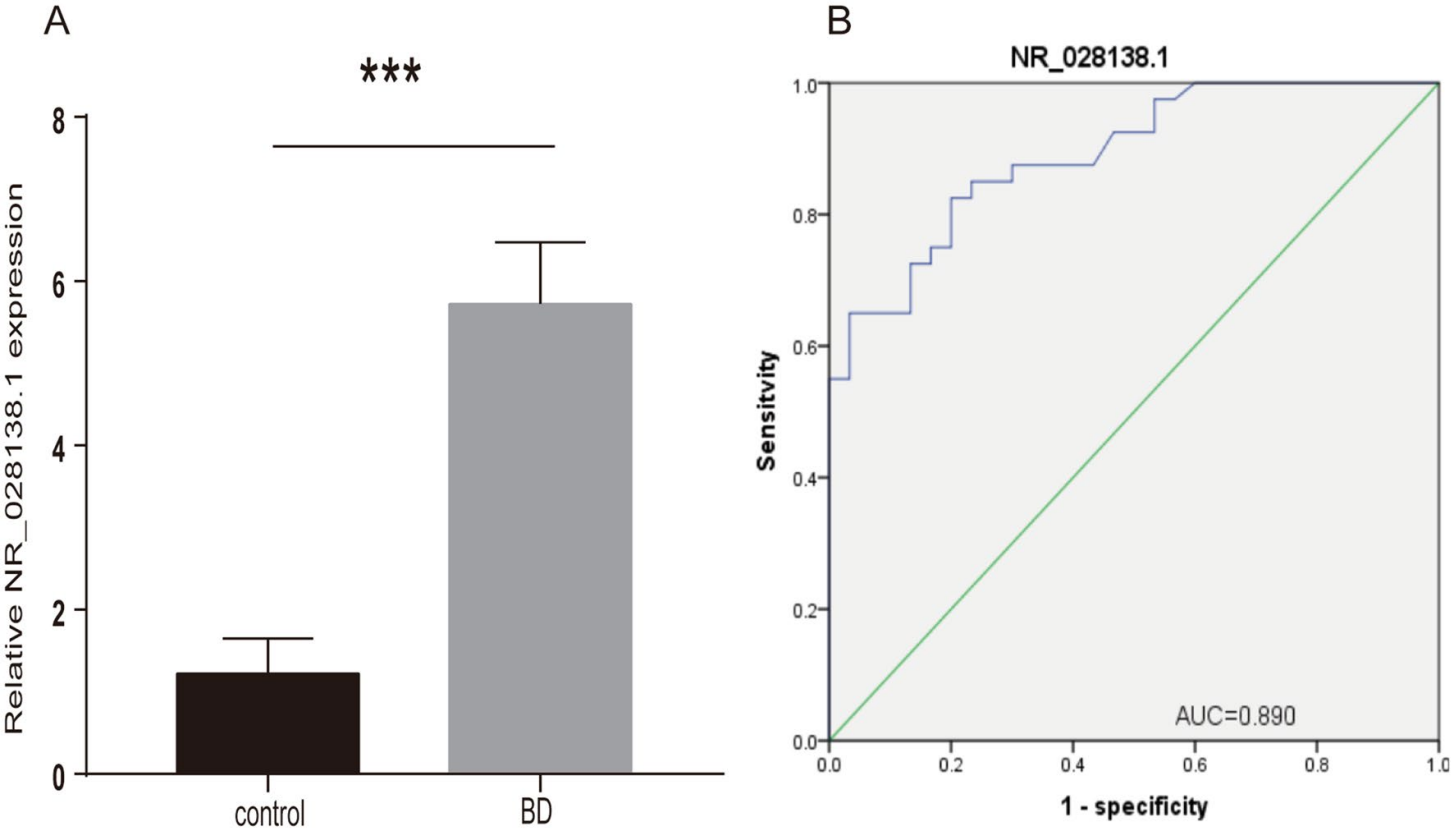
Second qRT-PCR validation of the data on NR_028138.1. (**A**) Relative expression levels of NR_028138.1. Black stars represent significant differences. (**B**) ROC analysis of an NR_028138.1-related model. *AUC* area under the curve.

NR_028138.1 was externally validated by qRT-PCR in another group of 146 volunteers (80 BD patients and 66 control subjects); the results indicated a significant upregulation in BD patients compared with that in the control subjects (*P* < 0.001) (Fig. 7A). NR_028138.1-related diagnostic model was further validated in these samples. AUC for NR_028138.1 was 0.890 (95% CI: 0.818–0.962; *P* < 0.0001), with sensitivity and specificity values of 90% and 85%, respectively (Fig. 7B).

## Discussion

The present study identified 576 significant DE-lncRNAs and 262 DE-mRNAs by high-throughput sequencing and confirmed that six RNAs (NR_028138.1, TCONS_00018621, TCONS_00002186, TNF, PID1, and SDK1) were significantly upregulated in BD patients compared with those in the control subjects. NR_028138.1 was correlated with BD, and AUC corresponding to a BD diagnostic model was 0.923 (*P* < 0.004, 95% CI: 0.830–0.999). We also constructed a ceRNA regulatory network to generate a hypothesis for pathogenesis of BD. NR_028138.1 was identified as a hub gene that participates in BD pathogenesis and can serve as a potentially useful diagnostic tool.

Recently, certain involvement of various lncRNAs, such as lnc-MALAT1 and apoptosis-related lncRNAs (CCAT2, TUG1, PANDA, NEAT1, FAS-AS1, and OIP5-AS1), was reported in BD^18–22^. However, these studies did not focus on the role of lncRNAs in the pathogenesis of BD. LncRNAs are transcripts with a length of more than 200 nucleotides that play a complex regulatory role in gene expression by acting as guides or decoys for other RNAs or by directly binding to DNA^23,24^. The results of the present study demonstrated that NR_028138.1 was expressed at a higher level in the BD group compared with that in the control group. Furthermore, ROC curve analysis showed that AUC for NR_028138.1 was 0.92 (90.3% sensitivity and 98% specificity). This diagnostic model was confirmed in 70 individuals in the second step of the validation and had AUC 0.890 (95% CI: 0.818–0.962; *P* < 0.0001). RNA sequencing data and miRNA target prediction software miRDB and TargetScan were used to construct a ceRNA regulatory network to determine potential functions of NR_028138.1. These results suggested that NR_028138.1 was significantly and independently associated with BD.

NR_028138.1 was coexpressed with 15 DE-mRNAs (SDK1, TNF, SGK1, CYP26B1, ABO, PITPNM3, GRB10, TNFRSF21, CEACAM1, PID1, DUSP13, SH3PXD2B, TIMP3, TFCP2L1, LRP2BP, and SP6). Overall, high PCCs between these RNAs represented a high possibility of similar functions. BD is known to be associated with changes in neural plasticity and neuronal survival. These processes are influenced by many factors, including synergistic effects of neurotransmitters, hormones, neurotrophic factors, and inflammatory mediators. Vares et al. showed an increase in the levels of inflammatory chemokines, including tumor necrosis factor (TNF)-α, interleukin (IL)-1β, and IL8, in euthymic BD patients compared with those in the healthy control subjects^25,26^. TNF-α is a cytokine composed of 157 amino acids, is produced after injury in macrophages, lymphocytes, neutrophils, and structural cells, and is stimulated by inflammation or infection. TNF-α is considered a proinflammatory molecule that enhances the immune response and helps to accelerate elimination of the pathogens. TNF-α can induce apoptosis in combination with its receptor TNFR1 in neurons and microglia. The number of glial cells in the prefrontal cortex is significantly decreased in bipolar patients. Additional necrosis and apoptosis are observed in oligodendrocytes of the frontal cortex and caudate nucleus of bipolar patients. These phenomena may be related to increased expression of TNF in the central nervous system of BD patients. Therefore, TNF is an important molecular target in the pathogenesis of BD. NR_028138.1 was colocalized with TNF, indicating that NR_028138.1 may participate in the development of BD by regulating the expression of TNF. Specific regulatory mechanism of this process needs further experimental verification.

LncRNAs can be used as miRNA sponges to regulate protein-coding RNAs during the course of various diseases, such as schizophrenia, depression, and anxiety disorder. A ceRNA network for BD was constructed in the present study based on 20 lncRNAs, 39 mRNAs, 62 miRNAs, and 71 lncRNA-miRNA-mRNA pathways. lncRNAs and mRNAs were coexpressed in each ceRNA pathway and interacted with same miRNAs. In this network, NR_028138.1 participated in 19 ceRNA pathways, which contained 12 miRNAs and 15 mRNAs, and one of the pathways was NR_028138.1 → hsa-miR-5196-5p → TNF. Hence, we hypothesize that NR_028138.1 regulates TNF by binding to hsa-miR-5196-5p.

Some advantages and limitations of the present study should be acknowledged. First, we identified DE-lncRNAs in BD patients versus the control subjects and comprehensively analyzed a ceRNA network involved in BD. Second, NR_028138.1 can be used as a diagnostic marker for BD, and robustness of the diagnostic model was verified externally. Third, we used bioinformatics to determine genes and networks that cause the disease. The limitations are as follows: First, we sampled peripheral blood lncRNAs but did not collect the samples from the central nervous system. Second, although we strictly controlled for other mental disorders, we did not control for common diseases, such as diabetes, hypertension, and metabolic diseases, which could have influenced the correlations between lncRNAs and BD. Third, the number of the samples, particularly in the BD group, limited statistical power of microarray analysis. Fourth, the present study focused only on the regulatory mechanism of ceRNA; however, lncRNA-related regulation is complex. Therefore, future studies should analyze all DE-lncRNAs in a larger cohort and explore other regulatory mechanisms. Finally, the results of the bioinformatic analysis should be verified in in vivo and in vitro experiments.

In conclusion, the present study confirmed that the level of NR_028138.1 in peripheral blood of patients with BD was significantly higher than that in the normal control subjects, and NR_028138.1 was identified as a potential biomarker of BD. Additionally, we successfully constructed a lncRNA-miRNA-mRNA ceRNA network, which was helpful to elucidate the pathogenesis of BD and crosstalk between lncRNAs and mRNAs.
